# Geldanamycin attenuates 3-nitropropionic acid-induced apoptosis and JNK activation through the expression of HSP 70 in striatal cells

**DOI:** 10.3892/ijmm.2014.1747

**Published:** 2014-04-22

**Authors:** YONG-JOON CHOI, NAM HO KIM, MAN SUP LIM, HEE JAE LEE, SUNG SOO KIM, WANJOO CHUN

**Affiliations:** Department of Pharmacology, College of Medicine, Kangwon National University, Chuncheon, Gangwon 200-701, Republic of Korea

**Keywords:** Huntington’s disease, geldanamycin, heat shock protein 70, 3-nitropropionic acid, reactive oxygen species

## Abstract

Although selective striatal cell death is a characteristic hallmark in the pathogenesis of Huntington’s disease (HD), the underlying mechanism of striatal susceptibility remains to be clarified. Heat shock proteins (HSPs) have been reported to suppress the aggregate formation of mutant huntingtin and concurrent striatal cell death. In a previous study, we observed that heat shock transcription factor 1 (HSF1), a major transcription factor of HSPs, significantly attenuated 3-nitropropionic acid (3NP)-induced reactive oxygen species (ROS) production and apoptosis through the expression of HSP 70 in striatal cells. To investigate the differential roles of HSPs in 3NP-induced striatal cell death, the effect of geldanamycin (GA), an HSP 90 inhibitor, was examined in 3NP-stimulated striatal cells. GA significantly attenuated 3NP-induced striatal apoptosis and ROS production with an increased expression of HSP 70. Triptolide (TL), an HSP 70 inhibitor, abolished GA-mediated protective effects in 3NP-stimulated striatal cells. To understand the underlying mechanism by which GA-mediated HSP 70 protects striatal cells against 3NP stimulation, the involvement of various signaling pathways was examined. GA significantly attenuated 3NP-induced c-Jun N-terminal kinase (JNK) phosphorylation and subsequent c-Jun phosphorylation in striatal cells. Taken together, the present study demonstrated that GA exhibits protective properties against 3NP-induced apoptosis and JNK activation via the induction of HSP 70 in striatal cells, suggesting that expression of HSP 70 may be a valuable therapeutic target in the treatment of HD.

## Introduction

Huntington’s disease (HD) is an autosomal dominant neurodegenerative disorder caused by an abnormal polyglutamine expansion within the protein huntingtin (1). Despite similar expression levels throughout the brain, mutant huntingtin selectively targets striatal neurons, with the cerebral cortex being affected later in the disease (2). Although selective striatal cell death is a prominent feature of HD, the underlying mechanism of striatal susceptibility remains to be clarified. Mitochondrial dysfunction has been reported to contribute to the pathogenesis of HD (3) and may underlie the selective neuronal degeneration (4).

Administration of 3-nitropropionic acid (3NP) in rodents and non-human primates has provided useful experimental models for HD (5,6). 3NP is an irreversible inhibitor of the mitochondrial complex II, which causes energy impairment and replicates most of the clinical and pathophysiological characteristics of HD, including spontaneous choreiform and dystonic movements, as well as the selective degeneration of striatum (6,7). 3NP has been reported to trigger the generation of superoxide radicals, secondary excitotoxicity and apoptosis (7,8). It has been reported that the c-Jun N-terminal kinase (JNK)/c-Jun signaling pathway plays an important role in 3NP-induced striatal degeneration (5).

Heat shock proteins (HSPs) are considered important protective effectors against a variety of cellular stresses (9,10). HSPs suppress protein misfolding by assisting misfolded proteins in the refolding process. For example, the overexpression of HSP 70 reduces the toxic accumulation of abnormal polyglutamine proteins and suppresses cell death in a variety of cellular models of polyglutamine diseases including HD (7,11–13). In addition, HSP 70 has been reported to block several steps of the apoptotic cascade such as upstream from mitochondria, release of cytochrome *c* and apoptosis-inducing factor (AIF), nuclear import of AIF, activation of procaspase-9 and -3, and even downstream of active caspase-3 (10,14–19).

Geldanamycin (GA) is a benzoquinone ansamycin antibiotic that inhibits the function of HSP 90 by binding to the ADP/ATP-binding pocket of the protein (20). HSP 90 client proteins play important roles in the regulation of the cell cycle, cell growth, survival, apoptosis, angiogenesis and oncogenesis (20). HSP 90 is a major repressor of the heat shock transcription factor 1 (HSF1), a major transcription factor of HSPs (21). Upon binding to HSP 90, GA induces the expression of HSP 70 through the action of HSF1 (21,22). It has been reported that GA activates a heat shock response and inhibits huntingtin aggregation in a cell culture model of HD (23).

The present study was conducted to examine whether GA attenuated 3NP-induced striatal damage and the underlying mechanism involved. GA exhibited an increased expression of HSP 70 and significantly suppressed 3NP-induced apoptosis, reactive oxygen species (ROS) generation, and JNK activation.

## Materials and methods

### Cell culture

The immortalized striatal progenitor cell line (STHdh^Q7^), which expresses endogenous wild-type huntingtin, was obtained from Dr Marcy E. MacDonald and maintained in Dulbecco’s modified Eagle’s medium (DMEM) supplemented with 10% (v/v) FBS, 10 U/ml of penicillin (all from Gibco, Invitrogen, Carlsbad, CA, USA) at 33°C in humidified air with 5% CO_2_.

### Cell viability assay

Striatal cells were plated in 6-well culture plates (Greiner Bio-One Inc., Longwood, FL, USA) and incubated at 33°C under 5% CO_2_, and 95% humidified air incubator. The cells were incubated with GA for 4 h prior to treatment with 3NP for another 24 h. After washing with PBS, 0.6 mg/ml 3-(4,5-dimethylthiazol-2-yl)-2,5-diphenyltetrazolium bromide (MTT) was added (100 μl/well) and incubated for 2 h at 33°C. MTT solution (40 μl) was then removed from each well and replaced with 500 μl of dimethyl sulfoxide (DMSO). The plates were incubated for 1 h at 33°C. Absorbance readings were taken at 570 nm using a Multiskan Ex microtitre plate reader (Thermo Fisher Scientific, Inc., Waltham, MA, USA). Data are expressed as % MTT reduction compared to a 100% signal from non-transfected cells.

### Lactate dehydrogenase leakage (LDH) assay

Striatal cells were exposed to 3NP (10 μM) overnight at 33°C for 24 h. After exposure to 3NP and GA, the medium was centrifuged at 250 × g for 10 min to harvest the cell culture media and the cell-free supernatant was obtained for the LDH activity assay using a commercial LDH detection kit (Roche Diagnostics Gmbh Mannheim, Germany) according to the manufacturer’s instructions.

### Western blotting

Striatal cells were washed with PBS three times and lysed by PRO-PREP protein extraction solution (Intron Biotechnology, Inc., Gyeonggi, Korea), and sonicated on ice. Protein concentrations of the homogenates were measured using the BCA method (Sigma-Aldrich, St. Louis, MO, USA) and diluted to a final concentration of 2 mg/ml with 2× reducing stop buffer (0.25 M Tris-HCl, pH 6.8, 5 mM EDTA, 5 mM EGTA, 25 mM dithiothreitol, 2% SDS, 10% glycerol, and bromophenol blue as the tracking dye). Equal amounts of proteins were separated on 8–12% SDS-polyacrylamide gels and transferred to a Hybond PVDF transfer membrane (GE Healthcare, Amersham, UK). The membranes were blocked in 5% skim milk in TBST (20 mM Tris-HCl, pH 7.6, 137 mM NaCl, 0.05% Tween-20) for 30 min at room temperature and sequentially incubated with an appropriate antibody; anti-HSP 90 monoclonal antibody (1:1,000), anti-HSP 70 monoclonal antibody (1:1,000) (both from Stressgen Biotechnologies, Victoria, BC, Canada), anti-cleaved caspase-3 and total caspase-3 polyconal antibody (1:1,000), anti-Cleaved PARP and total PARP polyconal antibody (1:1,000) (Cell Signaling Technology, Inc., Danvers, MA, USA), anti-IκB-α monoclonal antibody (Santa Cruz Biotechnology Inc., Santa Cruz, CA, USA), total JNK and P-JNK polyconal antibody (1:1,000), anti-c-Jun and P-c-Jun polyconal antibody (1:1,000) (both from Cell Signaling Technology, Inc.) and β-actin monoclonal antibody (1:2,500; Sigma-Aldrich) in the same buffer overnight at 4°C. After thoroughly washing with TBST, the membranes were washed three times with TBST and incubated with HRP-conjugated goat anti-rabbit IgG for polyclonal antibodies, or with HRP-conjugated goat anti-mouse IgG (1:2,500; Jackson ImmunoResearch Laboratories, West Grove, PA, USA) for 2 h at room temperature. The membranes were rinsed three times for 30 min with TBST, followed by four quick rinses with distilled water, and developed with the enhanced chemiluminescence method (GE Healthcare).

### FACS assay

Striatal cells were collected with a cell scraper, washed twice with cold PBS and then resuspended in 1× binding buffer at a concentration of 1×10^6^ cells/ml. One hundred microliters of the solution (1×10^5^ cells) were transferred to a 5 ml culture tube and 5 μl of Annexin V-PE and 5 μl of 7-AAD were added. The mixture was gently vortexed, incubated for 15 min at room temperature in the dark, and 400 μl of binding buffer was added to each tube. The stained cells were analyzed by flow cytometry (BD Model FACScan, BD Biosciences, Franklin Lakes, NJ, USA).

### DAPI staining

For DNA fragmentation studies, striatal cells were cultured in 6-well culture plates and treated with 3NP, GA and GA+3NP for 24 h. After washing with PBS, the striatal cells were fixed using 1% paraformaldehyde in PBS for 20 min, permeabilized using 0.1% Triton X-100 for 5 min, and stained with DAPI (1 μg/ml) (Invitrogen Life Technologies, Carlsbad, CA, USA) for 20 min. All steps were carried out at room temperature. Representative data were obtained by using confocal microscopy (Carl Zeiss, Inc., Thornwood, NY, USA).

### Measurement of intracellular ROS production

The striatal cells were cultured on 18 mm round coverslip at 33°C, 5% CO_2_. After 1 day, the coverslips were transferred into the 12-well plates and the media were changed (no phenol red media) for 1 h stabilization. After washing, each sample was stained by 10 μM H2DCFDA (H2DCFDA, Molecular Probe cat. no. D-399, stocked 50 mM in DMSO) for 10 min under culture conditions. The coverslips were washed three times with DMEM (no phenol red). Coverslips were located in the chamber, and media were added, and cells were immediately observed by confocal laser scanning microscopy (Olympus, Tokyo, Japan).

### Immunocytochemistry

The effect of 3NP into the nuclear translocation of NF-κB was examined by immunofluorescence assay using confocal microscopy. Following treatment, the cells were fixed with 4% PFA diluted in PBS for 20 min at room temperature and incubated for 10 min with 0.1% Triton X-100. After washing with PBS, the plates were preincubated with PBS, prior to incubation with 10% normal goat serum for 1 h to reduce the background at room temperature, followed by incubation with the anti-NF-κB antibody in PBS containing 10% normal goat serum overnight at 4°C. The plates were rinsed and incubated with an anti-rabbit TRITC-conjugated antibody (Invitrogen Life Technologies) for 2 h at room temperature. After washing, the nuclei were stained with Hoechst 33258 (100 mM) (Invitrogen Life Technologies) for 10 min and mounted under coverslips at room temperature. Representative data were obtained by using confocal microscopy (Carl Zeiss, Inc.). The digitally stored images were combined and shown with the accompanying software and Adobe Photoshop 4.0.

### Statistical analysis

Data were presented as means ± SE obtained from at least three independent experiments. The statistical difference was analyzed by one-way ANOVA with Tukey’s post-hoc test using SPSS software 12K (SPSS, Inc., Chicago, IL, USA). P<0.05 was considered statistically significant.

## Results

### GA results in the increased expression of HSP 70 and attenuates 3NP-induced apoptosis in striatal cells

**Figure 1:**
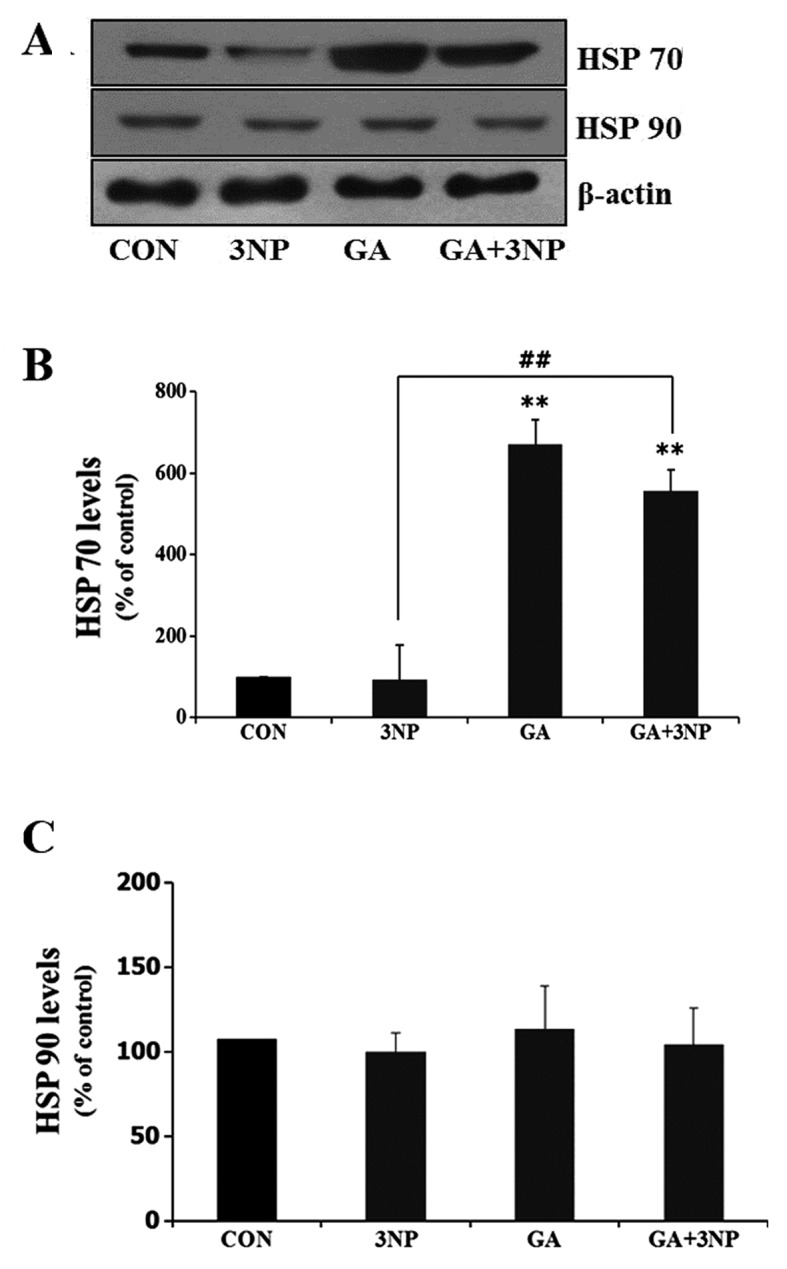
Geldanamycin (GA)-induced expression of heat shock protein (HSP) 70 in striatal cells. (A) Striatal cells were treated with 500 nM GA and incubated in the presence or absence of 10 μM 3-nitropropionic acid (3NP). After 24 h of incubation, HSP 70 and HSP 90 were assessed by immunoblotting. HSP 70 expression was increased by GA, whereas expression of HSP 90 did not change. Loading control was confirmed by β-actin. (B and C) Quantitative analysis was obtained from three individual experiments (n=3). ^**^P<0.01 indicates significant differences compared to the control. ^##^P<0.05 indicates significant differences between the indicated groups.

To examine the effect of GA, an HSP 90 inhibitor, in the expression of HSPs, the expression levels of HSP 70 and HSP 90 were examined. GA resulted in the increased expression of HSP 70 (Fig. 1A). However, the expression level of HSP 90 was not significantly changed with GA, which inhibits the function of HSP 90 by binding to the ADP/ATP-binding pocket of the protein.

**Figure 2:**
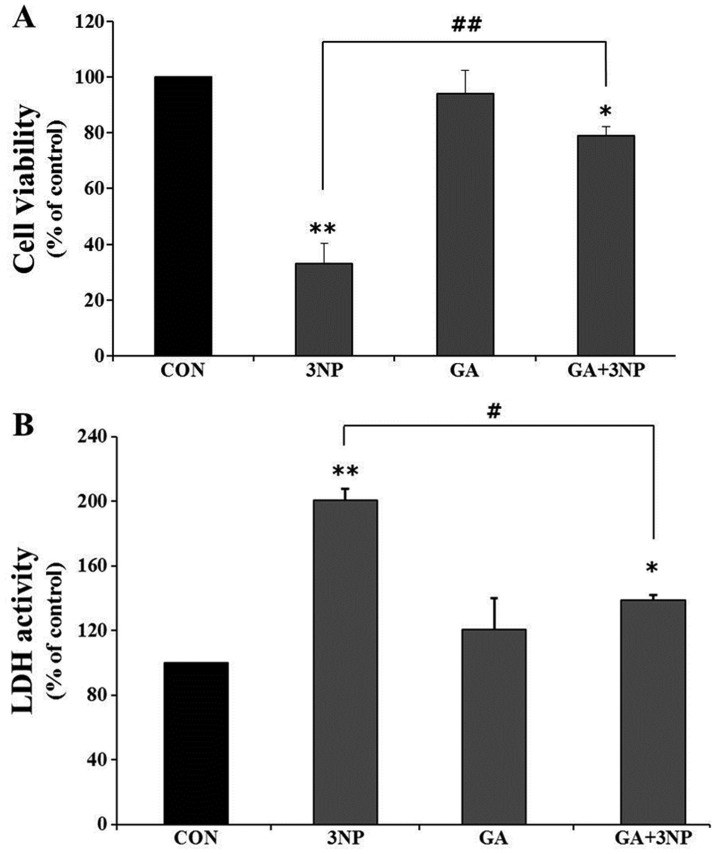

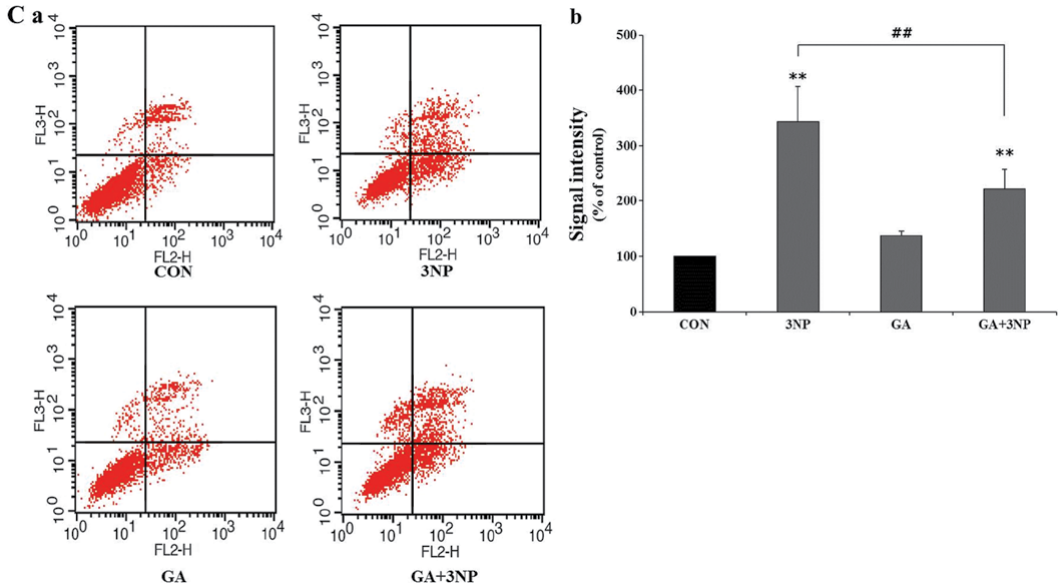
Protective effects of geldanamycin (GA) on the cytotoxicity of 3-nitropropionic acid (3NP). (A and B) Cell viability was examined with 3-(4,5-dimethylthiazol-2-yl)-2,5-diphenyltetrazolium bromide (MTT) assay and lactate dehydrogenase leakage (LDH) assay. Striatal cells were treated with 10 μM 3NP, 500 nM and their combination as indicated. After 24 h, MTT and LDH assays were performed. GA significantly attenuated 3NP-induced cytotoxicity in striatal cells, albeit not completely. Data are shown as mean ± SD from three individual experiments (n=3). (Ca) Representative FACS images and (Cb) quantitative analysis of FACS data. Striatal cells were incubated in the presence or absence of 3NP after treatment of GA. FACS assay was carried out to determine initiation and amplitude of apoptotic dell death. Only Annexin V-positive cells were indicated as early apoptotic cells and Annexin V- and 7-AAD-positive cells were considered as late apoptotic cells. The number of total apoptotic cells, which are positive to both Annexin-5 and 7-AAD, was significantly decreased in presence of GA compared to only 3NP. Data were obtained from four independent experiments (n=4). ^*^P<0.05 and ^**^p<0.01 indicate differences compared to the control. ^#^P<0.05 and ^##^p<0.01 indicates significant differences between the indicated groups.

In order to examine the effect of GA on the viability of 3NP-stimulated striatal cells, the cells were treated with 3NP in the absence or presence of GA. Significant striatal cell death was observed with 3NP treatment in MTT and LDH assays (Fig. 2A and B). However, GA significantly attenuated 3NP-induced striatal cell death. In addition, the number of positive cells of 7-AAD and FITC, which indicate dead cells, was significantly reduced with GA in the FACS analysis (Fig. 2Ca and b).

**Figure 3:**
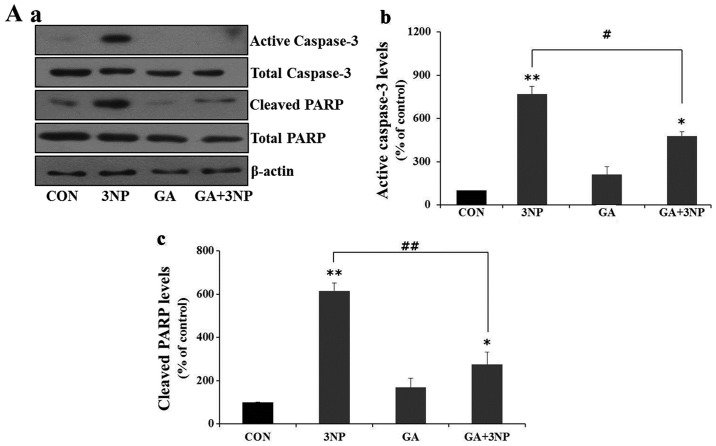

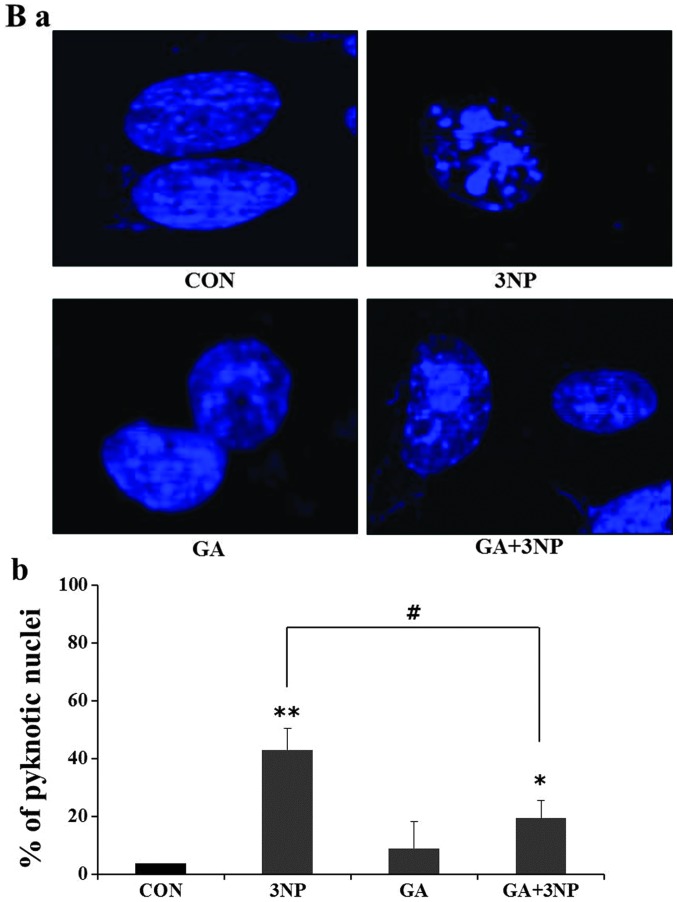
Effects of 3-nitropropionic acid (3NP) and geldanamycin (GA) on apoptosis. Striatal cells were treated with GA, 3NP and GA+3NP. (Aa) After 24 h of treatment, cells were collected and protein extracts prepared from each samples were analyzed by immunoblotting using indicated antibodies. GA suppressed 3NP-induced caspase-3 activation and level of cleaved PARP. β-actin was used as a loading control. (Ab and c) Quantitative analysis was obtained from three individual experiments (n=3). The nuclear morphological changes are shown by 3NP-treated striatal cells. (Ba) The abrogation of nuclear damage by GA was measured using DAPI staining and (Bb) quantitative analysis of apoptotic cells. The more apoptotic cells were observed in only 3NP treated striatal cells compared to GA+3NP-treated striatal cells. Apoptotic cells were quantitatively counted using a microscope. In only 3NP-170-treated striatal cells, ~42% of cells showed DNA fragmentation whereas ~21% of cells included fragmentation in GA+3NP-treated striatal cells. Approximately 100 DAPI-positive cells were counted in each experiment. Quantitative analysis was obtained from three individual experiments (n=3). ^*^P<0.05, ^**^p<0.01 indicate significant differences compared to the control. ^#^P<0.05, ^##^p<0.01 indicates significant differences between the indicated groups.

Given the fact that 3NP induces striatal cell death in the present study, we examined whether 3NP induces apoptosis and whether GA attenuated 3NP-induced striatal cell death. Treatment of 3NP resulted in the cleavage of caspase-3 and PARP, which indicate the activation of apoptosis (Fig. 3A). GA significantly attenuated 3NP-induced production of active caspase-3 and cleaved PARP in striatal cells (Fig. 3A). In addition, GA significantly reduced the number of 3NP-induced apoptotic nuclei (Fig. 3B).

### GA attenuates 3NP-induced ROS production

**Figure 4:**
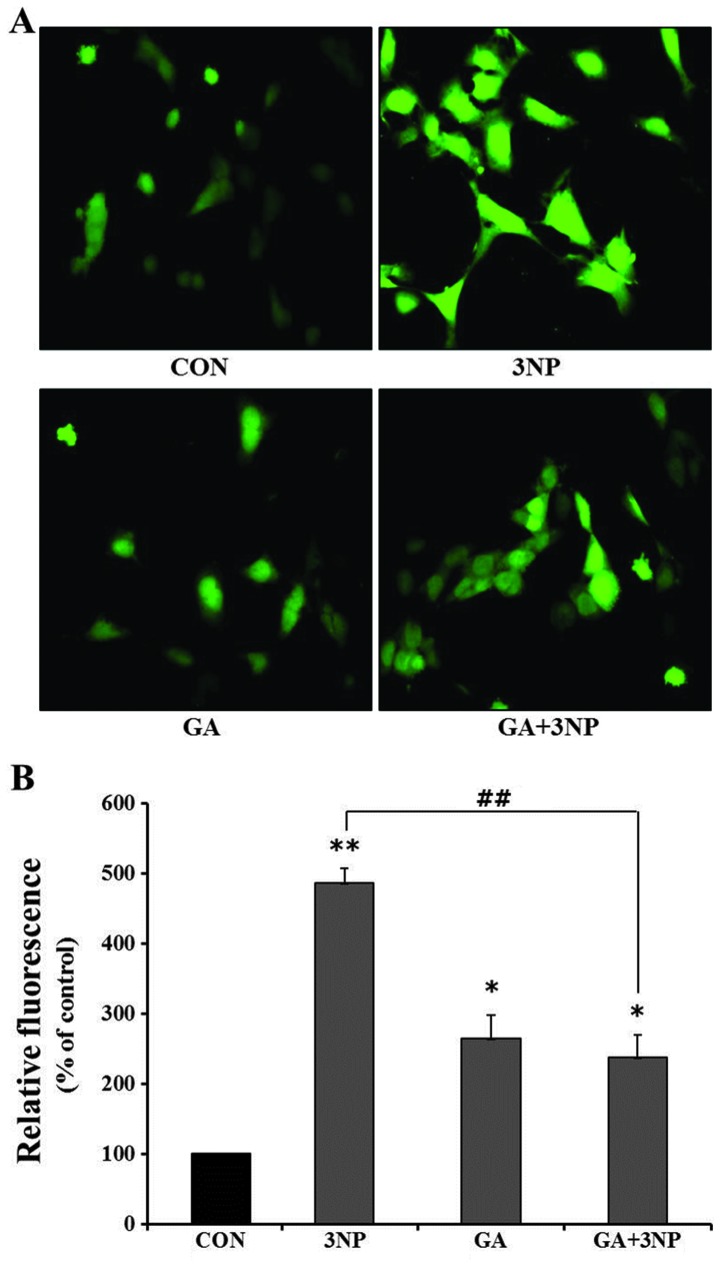
Geldanamycin (GA) significantly reduced intracellular reactive oxygen species (ROS) generation by 3-nitropropionic acid (3NP). (A) Intracellular level of ROS was measured using confocal microscopy. Striatal cells were treated with GA and incubated in the presence or absence of 3NP. Cells were incubated with fluorescence probe H2DCFDA (10 μM). (B) H2DCFDA fluorescence was quantitatively analyzed with Fluoview 300 software. The production of 3NP-induced ROS was significantly attenuated by GA in striatal cells. Results are the means ± SD of three independent experiments performed in triplicate. ^*^P<0.05, ^**^p<0.01 indicate significant differences compared to the control. ^##^P<0.05 indicates significant differences between the indicated groups.

We have previously reported that the overexpression of HSF1 significantly attenuated 3NP-induced ROS production in striatal cells (7). To investigate the effects of GA in 3NP-induced ROS production, the intracellular ROS generation was measured in the absence or presence of GA in 3NP-challenged striatal cells. Treatment of 3NP resulted in the production of a considerable amount of the intracellular ROS in striatal cells. GA significantly attenuated 3NP-induced ROS production, albeit not completely (Fig. 4). Fig. 4A shows a representative confocal image of intracellular level of ROS and Fig. 4B shows quantitative analysis of ROS production. The result demonstrates that GA protects cells by inhibiting the production of ROS in 3NP-challenged striatal cells.

### GA attenuates 3NP-induced IκB degradation and nuclear translocation of NF-κB

**Figure 5:**
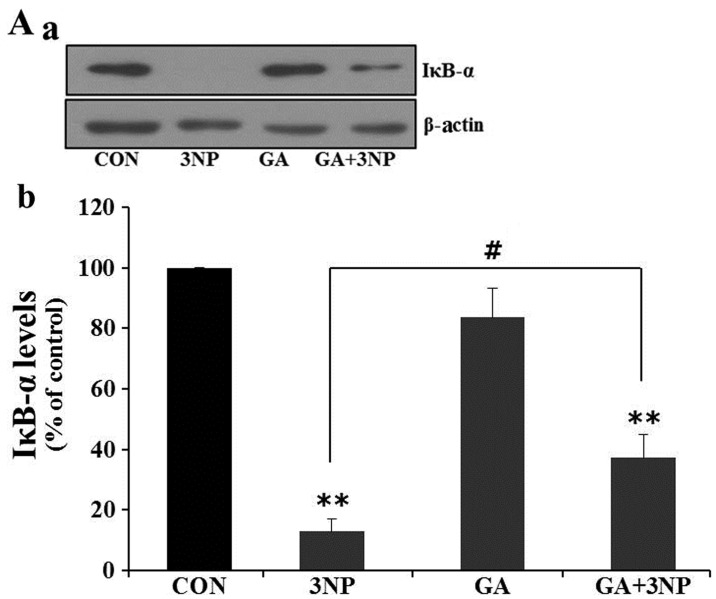

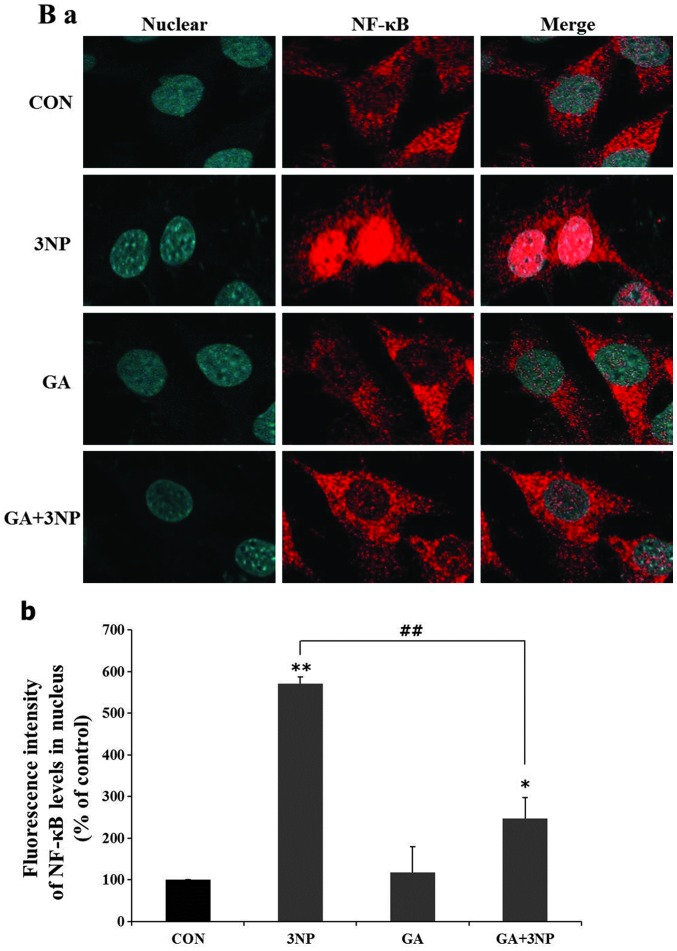
Geldanamycin (GA) inhibited 3-nitropropionic acid (3NP)-induced IκB degradation and nuclear translocation of NF-κB. Striatal cells were treated with GA, 3NP, GA+3NP or were untreated (control). (Aa) After 24 h of incubation, total proteins were extracted for immunoblotting of IκB-α. Degradation of IκB-α attenuated the GA+3NP group compared to the 3NP-only group. β-actin was used as a loading control. (Ba) Nuclear translocation of NF-κB was examined using immunocytochemistry assay. NF-κB (red panels) was mainly localized in the cytoplasm (control and GA). Nuclear translocation of NF-κB was facilitated only in 3NP compared to GA+3NP. Nuclei were visualized by Hoechst staining (Hoechst 33258). (Ab and Bb) Quantitative analysis was obtained from three individual experiments (n=3). ^*^P<0.05, ^**^p<0.01 indicate significant differences compared to the control. ^#^P<0.05, ^##^p<0.01 indicates significant differences between the indicated groups.

It has been reported that ROS facilitates cell death by inducing inflammatory responses via the activation of NF-κB-mediated transcription (24–26). IκB inhibits the nuclear translocation of NF-κB by retaining it in cytoplasm. Under stress conditions, IκB proteins are rapidly degraded by the proteasome, and released NF-κB translocates into the nucleus to activate apoptotic pathways. To examine whether GA has an impact on NF-κB transcription, the effects of GA on IκB degradation and the nuclear translocation of NF-κB were examined. Treatment of 3NP markedly depleted intracellular IκB in striatal cells. However, GA significantly attenuated 3NP-induced IκB degradation (Fig. 5A). To confirm whether the nuclear translocation of NF-κB was affected by change at the intracellular level of IκB, intracellular localization of NF-κB was examined with immunocytochemistry. Treatment of 3NP obviously increased the nuclear translocation of p65 subunit of NF-κB (Fig. 5B). Geldanamycin significantly attenuated the nuclear translocation of NF-κB in 3NP-challeged striatal cells (Fig. 5Ba and b).

### Triptolide (TL) abrogates GA-mediated HSP 70 expression

**Figure 6:**
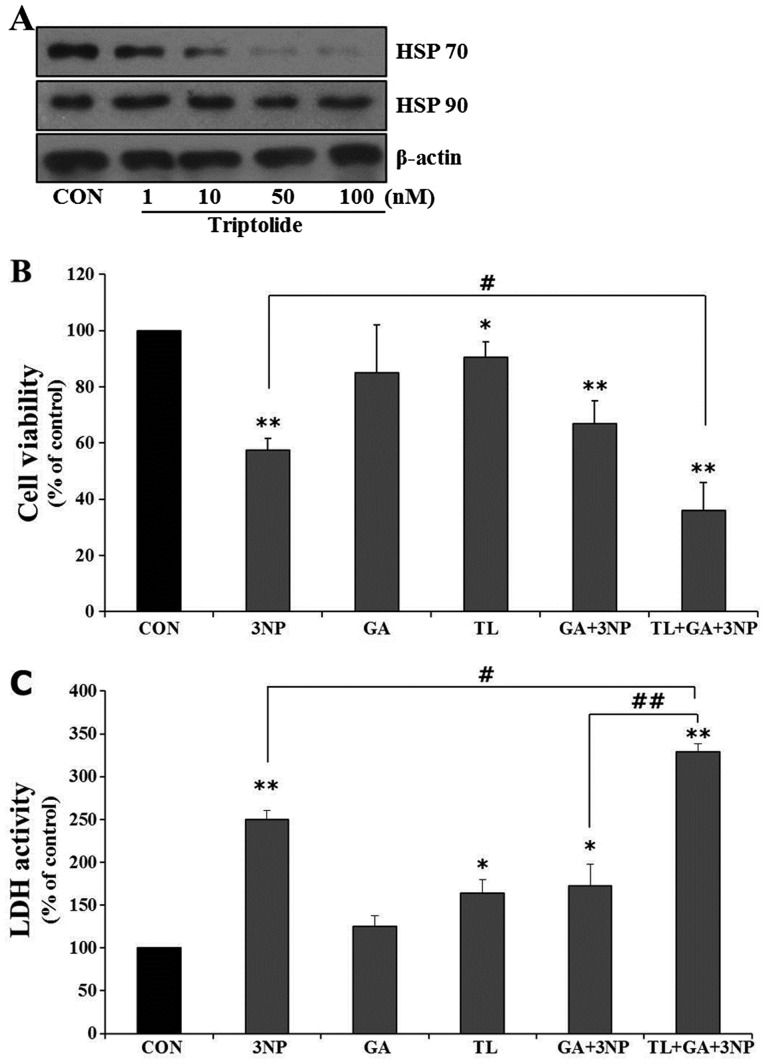
Triptolide (TL) inhibits the expression of heat shock protein (HSP) 70 and abrogates protective effect by geldanamycin (GA). (A) Striatal cells were treated with indicated concentration of TL. After 2 h, cells were harvested for immunoblotting of HSP 70 and HSP 90. Expression of HSP 70 was inhibited in a dose-dependent manner by TL, while no change was observed for HSP 90. β-actin was used as a loading control. TL significantly attenuated the protective effect of GA against 3-nitropropionic acid (3NP)-induced cell death. (B and C) Cell viability was examined with 3-(4,5-dimethylthiazol-2-yl)-2,5-diphenyltetrazolium bromide (MTT) assay and lactate dehydrogenase leakage (LDH) assay. Prior to treatment with 3NP, striatal cells were incubated for 2 h with TL, an inhibitor of HSP 70. TL did not cause cell death at a concentration of 50 nM used in the present study. Data were obtained from four independent experiments (n=4) and expressed as mean ± SD. ^*^P<0.05 and ^**^p<0.01 indicate significant differences compared to the control. ^#^P<0.05 and ^##^p<0.01 indicates significant differences between the indicated groups.

Given the hypothesis that HSP 70 plays an important role in GA-mediated cellular protection in 3NP-challenged striatal cells, the effect of GA was examined in the absence of HSP 70 expression. The depletion of HSP 70 expression was achieved with TL, which inhibits the endogenous HSP 70 gene expression (27). TL downregulated HSP 70 expression in a concentration-dependent manner in striatal cells without affecting HSP 90 expression (Fig. 6A). To investigate the role of TL in GA-mediated cellular protection, cell viability was examined. TL showed negligible cytotoxicity at a concentration range used in the present study. However, TL significantly abolished GA-mediated cellular protection against 3NP treatment (Fig. 6B and C). The results strongly suggested that HSP 70 plays an essential role in GA-mediated cellular protection in 3NP-challenged striatal cells.

### GA inhibits 3NP-induced JNK and c-Jun phosphorylation

**Figure 7:**
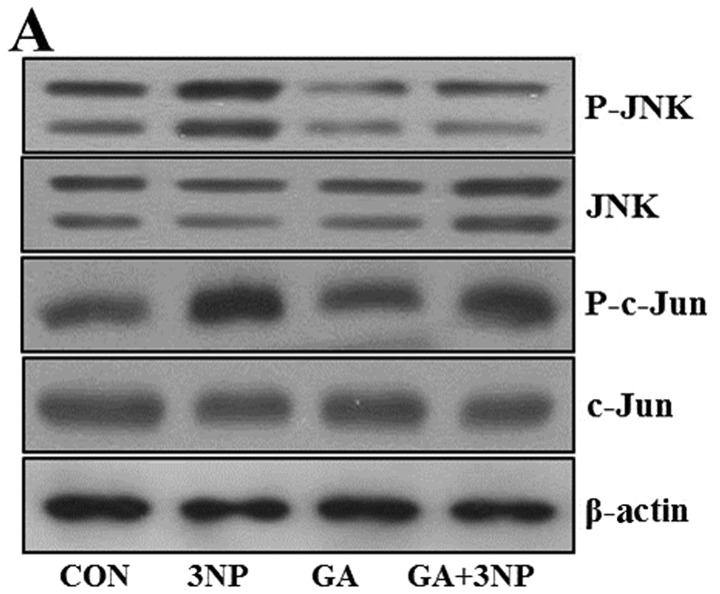

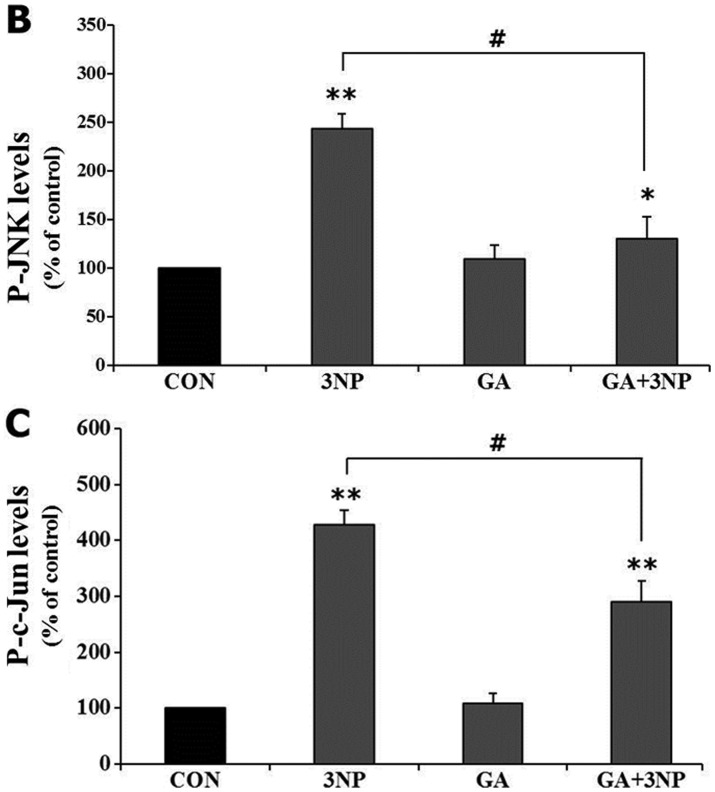
Geldanamycin (GA) inhibits the phosphorylation of c-Jun N-terminal kinase (JNK)/c-Jun via mediation of heat shock protein (HSP) 70. Striatal cells were treated with GA, 3-nitropropionic acid (3NP) and GA+3NP. (A) After 4 h of treatment, cells were harvested and total proteins were extracted for immunoblotting of JNK and c-Jun. Treatment of GA resulted in the significant suppression of phosphorylation of JNK/c-Jun by 3NP. β-actin was used as a loading control. (B and C) Quantitative analysis of immunoblots was obtained from three individual experiments (n=3). ^*^P<0.05, ^**^p<0.01 indicate significant differences compared to the control. ^#^P<0.05, ^##^p<0.01 indicates significant differences between the indicated groups.

It has been previously reported that activation of JNK plays a key role in 3NP-induced striatal neurodegeneration (5). Therefore, the effects of GA on 3NP-induced JNK activation and subsequent c-Jun phosphorylation were examined in the present study. Treatment of 3NP resulted in the activation of JNK (Fig. 7A). However, GA significantly attenuated JNK phosphorylation in 3NP-challenged striatal cells (Fig. 7A). In addition, GA significantly suppressed 3NP-induced c-Jun phosphorylation in striatal cells. The data strongly demonstrate that the JNK/c-Jun signaling pathway is an important molecular pathway in 3NP-induced striatal damage and that GA exerts a cellular protective effect through suppression of the JNK/c-Jun pathway.

## Discussion

The present study has demonstrated that GA significantly suppressed 3NP-induced apoptotic cell death and ROS generation through the expression of HSP 70 in a striatal cell model. GA also significantly attenuated the 3NP-induced activation of the JNK/c-Jun signaling pathway.

HSPs have been reported to attenuate protein aggregation and neurodegeneration in HD models (28,29). HSP 70 expression was increased in oxidative stress conditions, which is presumably considered a cellular compensatory mechanism against oxidative stresses (14,30–33). It has been reported that inducers of HSP 70 significantly suppressed toxicity of mutant huntingtin in a *C. elegans* model (34). Recently, we observed that the overexpression of HSF1 resulted in a significantly increased expression of HSP 70 and attenuated apoptosis in 3NP-challenged striatal cells (7). In addition, inhibition of HSP 70 function with methylene blue significantly abolished HSF1-mediated protection against 3NP-induced striatal damage (7), suggesting that HSP 70 plays a major role in HSF1-mediated anti-apoptotic action in striatal cells. In the present study, inhibition of HSP 70 gene expression with TL also significantly attenuated GA-mediated protection in striatal cells, confirming that HSP 70 plays an essential role in cellular protection in 3NP-challenged striatal cells.

HSP 90 is constitutively expressed in mammalian cells and plays an essential role in facilitating the proper folding, maturation, and activity of its client proteins (35), which eventually regulates a variety of cellular events including cell survival, apoptosis, and oncogenesis (20). HSP 90 associates with its client proteins in an ATP-dependent manner (36,37). GA specifically interferes with this association by occupying the ATP-binding pocket of HSP 90 and dissociates client proteins from the protein (38–40). HSP 90 has also been reported to be a major repressor of HSF1 (21). It has been reported that GA induces the expression of HSPs such as HSP 70 and HSP 40 and inhibits huntingtin aggregation in a cell culture model of HD (23). In that study, HSPs significantly attenuated the mutant huntingtin-induced toxicity and the number of mutant huntingtin aggregates. However, it has also been reported that HSPs did not affect the aggregate formation of mutant huntingtin (41), suggesting that HSPs may exert its protective actions independent of suppression of aggregate formation. Therefore, more studies are necessary to elucidate the exact mechanism by which HSPs exert cytoprotection in the pathogenesis of HD. In the present study, GA exhibited an increased expression of HSP 70 and a significant suppression of apoptotic cell death ROS production in 3NP-challenged striatal cells. GA has also been reported to protect against MPTP-induced dopaminergic neurotoxicity through the induction of HSP 70 in an animal model (42).

Mitochondrial dysfunctions have been reported to be involved in the pathogenesis of HD (43). Individuals with HD showed a decreased mitochondrial enzyme activities in the striatum (44,45) and mitochondria from HD patients were shown to be more sensitive to apoptosis (46). Mitochondrial toxin 3NP produces selective striatal lesions (47,48) and clinical features of HD such as choreiform movements and locomotor dysfunction (49,50). 3NP is known to induce striatal neurodegeneration via the activation of the JNK/c-Jun signaling pathway (5). Activation of JNK occurred progressively and selectively in the striatum and c-Jun activation was followed in the same striatal region (5). Furthermore, overexpression of the dominant negative form of c-Jun completely abolished 3NP-induced striatal neurodegeneration, indicating that the JNK/c-Jun signaling pathway is an important molecular event in 3NP-induced striatal degeneration (5). In the present study, significantly increased phosphorylation of JNK and c-Jun was observed with 3NP treatment in striatal cells, confirming that JNK/c-Jun signaling pathway is crucial in 3NP-induced striatal toxicity.

HSP 70 has been reported to block JNK activation and prevent apoptosis in response to protein-damaging and physiological stimuli (51–53). HSP 70 modulates the activity of JNK through direct binding to the protein (54) and that HSP 70 deficiency results in activation of the JNK signaling pathway (55). Recently, it has been reported that HSP 70 inhibits the JNK signaling pathway and subsequently prevents Bax-mediated apoptosis (56). In the present study, inhibition of HSP 70 gene expression with TL significantly abolished GA-mediated striatal survival against 3NP-induced cell death, suggesting that HSP 70 may be a major mediator for the suppression of JNK/c-Jun activation in striatal cells. However, more studies are necessary to clearly understand the mechanism by which geldanamycin-induced HSP 70 inhibits the JNK/c-Jun signaling pathway in 3NP-challenged striatal cells.

Taken together, the present study clearly demonstrates that GA exerts anti-apoptotic properties such as suppression of apoptosis and JNK/c-Jun signaling in 3NP-challenged striatal cells presumably through the expression of HSP 70, suggesting that GA may be a valuable therapeutic agent to increase the intracellular level of HSP 70, which plays a beneficial role in the pathogenesis of HD.
